# Social Media Use and Mental Health Among Hispanic/Latinx Dementia Caregivers

**DOI:** 10.1093/geroni/igaf122.1703

**Published:** 2025-12-31

**Authors:** Sofia Mildrum Chana, Natashia Bibriescas, Andres Azuero, Michael Crowe, Frank Puga

**Affiliations:** The University of Alabama at Birmingham, Birmingham, Alabama, United States; University of Alabama at Birmingham, Birmingham, Alabama, United States; University of Alabama at Birmingham, Birmingham, Alabama, United States; University of Alabama at Birmingham, Birmingham, Alabama, United States; University of Alabama at Birmingham, Birmingham, Alabama, United States

## Abstract

Loneliness and poor mental health are commonly observed among Hispanic & Latinx (H&L) family dementia caregivers given caregiving-related stress, frequent reliance on informal caregiving, and resource underutilization. Given the ubiquitous presence of social networking sites (SNS, i.e., social media), caregivers may turn to these platforms for coping and social support. However, no research has yet evaluated the impact of SNS use on caregivers’ mental health. Therefore, this study examines variations in daily SNS use and mental health experiences of H&L caregivers employing daily diary surveys from an ongoing national level study entitled “Nuestros Días (Our Days).” Participants (n = 80) primarily identified as female (85.0%) with an average age of 56.86 years (SD = 9.94) and preferred Spanish language (82.7%). About 68.3% of caregivers had been providing care for three or more years to their biological parent (60.1%) and reported using SNS an average of 152.53 minutes (SD = 69.66) daily. Data were analyzed using multilevel modeling where days were nested in people. Two separate models were conducted where within-person variations in hours of daily passive (i.e., consumption of SNS content without active engagement) and active SNS use (i.e., engagement with content and deliberate interaction with other users) predicted daily depressive and anxiety symptoms. A significant positive association was observed between caregivers’ daily passive SNS use and depressive symptoms (B = 0.75, p<.001) and anxiety symptoms (B = 0.66, p<.01). However, there were no significant associations between daily active SNS use and depressive or anxiety symptoms. Findings highlight the detrimental contributions of passive SNS use to caregivers’ mental health.

